# Radiomics for detecting prostate cancer bone metastases invisible in CT: a proof-of-concept study

**DOI:** 10.1007/s00330-021-08245-6

**Published:** 2021-09-24

**Authors:** Ricarda Hinzpeter, Livia Baumann, Roman Guggenberger, Martin Huellner, Hatem Alkadhi, Bettina Baessler

**Affiliations:** 1 Institute of Diagnostic and Interventional Radiology, University Hospital Zurich, University of Zurich, Raemistr. 100, CH-8091 Zurich, Switzerland; 2 Department of Nuclear Medicine, University Hospital Zurich, University of Zurich, Zurich, Switzerland

**Keywords:** Computed tomography, Prostate cancer, Bone metastases, Texture analysis, Radiomics

## Abstract

**Objectives:**

To investigate, in patients with metastatic prostate cancer, whether radiomics of computed tomography (CT) image data enables the differentiation of bone metastases not visible on CT from unaffected bone using ^68^ Ga-PSMA PET imaging as reference standard.

**Methods:**

In this IRB-approved retrospective study, 67 patients (mean age 71 ± 7 years; range: 55–84 years) showing a total of 205 ^68^ Ga-PSMA-positive prostate cancer bone metastases in the thoraco-lumbar spine and pelvic bone being invisible in CT were included. Metastases and 86 ^68^ Ga-PSMA-negative bone volumes in the same body region were segmented and further post-processed. Intra- and inter-reader reproducibility was assessed, with ICCs < 0.90 being considered non-reproducible. To account for imbalances in the dataset, data augmentation was performed to achieve improved class balance and to avoid model overfitting. The dataset was split into training, test, and validation set. After a multi-step dimension reduction process and feature selection process, the 11 most important and independent features were selected for statistical analyses.

**Results:**

A gradient-boosted tree was trained on the selected 11 radiomic features in order to classify patients’ bones into bone metastasis and normal bone using the training dataset. This trained model achieved a classification accuracy of 0.85 (95% confidence interval [CI]: 0.76–0.92, *p* < .001) with 78% sensitivity and 93% specificity. The tuned model was applied on the original, non-augmented dataset resulting in a classification accuracy of 0.90 (95% CI: 0.82–0.98) with 91% sensitivity and 88% specificity.

**Conclusion:**

Our proof-of-concept study indicates that radiomics may accurately differentiate unaffected bone from metastatic bone, being invisible by the human eye on CT.

**Key Points:**

*• This proof-of-concept study showed that radiomics applied on CT images may accurately differentiate between bone metastases and metastatic-free bone in patients with prostate cancer.*

*• Future promising applications include automatic bone segmentation, followed by a radiomics classifier, allowing for a screening-like approach in the detection of bone metastases.*

**Supplementary Information:**

The online version contains supplementary material available at 10.1007/s00330-021-08245-6.

## Introduction

Prostate cancer (PCa) is the most common solid tumor in men, accounting for the third most common cause of cancer-associated death in developed countries [1]. Metastatic spread of PCa is primarily to the skeleton, with pelvis, vertebra, and ribs being the most commonly affected bones [2, 3]. Approximately 12% of patients present with bone metastases at the time of diagnosis [2], resulting in restricted quality of life with a 5-year survival rate of only 6% [4–6]

Computed tomography (CT) is a useful and widely available imaging tool for screening the skeleton, allowing to establish the extent of cortical bone destruction, the presence of pathological fractures and to plan operative treatment [7, 8]. However, CT has shortcomings in detecting small bone metastases without apparent pathologic changes of the osseous structure and—if at all—showing only very subtle imaging findings [9, 10]. Positron emission tomography (PET) in combination with CT (PET/CT) using ^68^ Ga-PSMA as radiotracer has emerged as a useful technique for the diagnosis of bone metastases in patients with PCa, showing robust performance, with a patient-based sensitivity and specificity of 98.7–100% and 88.2–100%, respectively [11–13]. Interestingly, ^68^ Ga-PSMA often demonstrates bone metastases while CT shows no visible abnormality [14], when CT images are being analyzed in a pure qualitative manner.

Recent developments of machine learning techniques and the huge growth of computational power has driven the field of radiomics [15]. The principles of radiomics include extraction of high-dimensional data from various sources of medical images, followed by an analysis of various classes of radiomic features, aiming to support clinical decision-making and overcoming the limitations of a solely visual image interpretation [16]. Several studies have already shown that a radiomics-based machine learning method allows not only for the quantification of imaging results but has the potential to detect pathological findings in the absence of visible abnormalities [16–18]. We hypothesized that the application of CT-derived radiomics in metastatic bone disease from prostate cancer might reveal important imaging information that cannot be detected visually by the human eye.

Thus, the aim of this proof-of-concept study was to investigate, in patients with metastatic prostate cancer, whether radiomics of CT image data enables the differentiation of bone metastases invisible on CT from unaffected bone.

## Methods

**Fig. 1:**
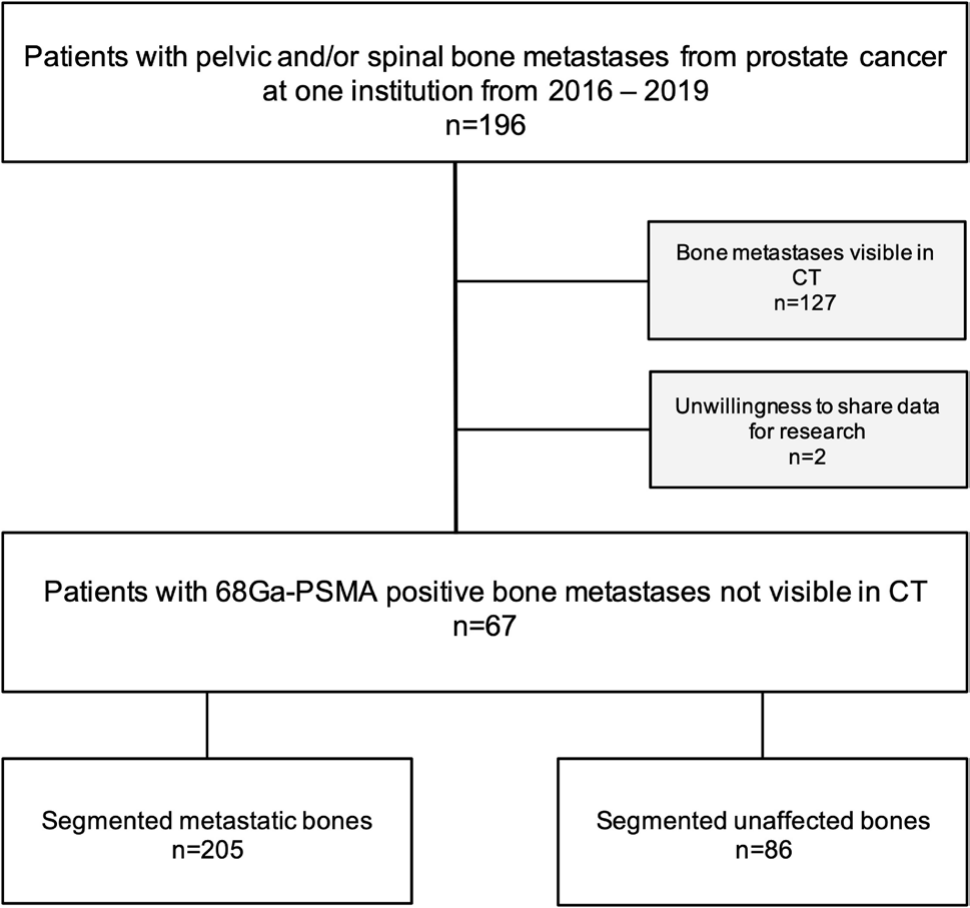
Flowchart of the study cohort

Based on a database search, 196 patients with ^68^ Ga-PSMA-positive bone metastases in the thoracic and/or lumbar spine and/or pelvic bones who underwent imaging in one tertiary referral center between May 2016 and June 2019 were identified. A senior radiologist with 10 years of experience in oncologic imaging excluded 127 of these 196 patients (65%) because the bone metastases were also clearly visible on CT. Two patients denied usage of their medical data for research (Fig. 1). Finally, 67 patients (mean age 71 ± 7 years; range: 55–84 years) were included. At the time of the study, the indication of PSMA PET/CT was biochemical recurrence of prostate cancer with a rise of the PSA ≥ 0.2 ng/mL following radical prostatectomy, or a rise of 2 ng/mL or more above the nadir PSA after radiation therapy. Nowadays, under special circumstances, PSMA PET/CT is also used and will be reimbursed in patients with initial diagnosis of prostate cancer.

Demographic patient data and anatomic locations of all segmented bone volumes are provided in Tables 1 and 2, respectively. Our study had institutional review board and local ethics committee approval. All patients provided written informed consent prior to the study.

**Table 1:** Demographic data of the study cohort

	Study cohort (*n* = 67)
Age (years) (mean ± SD)	71 ± 7
Weight (kg) (mean ± SD)	82 ± 12
Height (cm) (mean ± SD)	175 ± 5
BMI (kg/m^2^) (mean ± SD)	26 ± 3
TNM stage (initial)	
T1 T2 T3 T3a (extracapsular extension) T3b (infiltration of seminal vesicles) T4	5 (7%) 16 (24%) 38 (57%) 21 (55%) 17 (45%) 8 (12%)
Gleason Score	
Grade 1 (3 + 3 = 6) Grade 2 (3 + 4 = 7a) Grade 3 (4 + 3 = 7b) Grade 4 (4 + 4 = 8) Grade 5 (Gleason 9–10) Initial PSA (ng/ml) (mean ± SD)	2 (3%) 12 (18%) 14 (21%) 8 (12%) 31 (46%) 72 ± 172
Treatment	
*Surgery* Radical prostatectomy Radical prostatovesiculectomy Radical prostatovesiculectomy with pelvic lymphadenectomy	50 (75%) 13 (19%) 10 (15%) 27 (40%)
*Radiation and/or hormone therapy*	67 (100%)

**Table 2:** Anatomic locations of affected and unaffected segmented bone volumes

	Affected bone volumes	Unaffecetd bone volumes
Thoracic spine	*n* = 81	*n* = 22
Lumbar spine	*n* = 46	*n* = 59
Left iliac bone	*n* = 28	*n* = 3
Right iliac bone	*n* = 24	*n* = 2
Sacrum	*n* = 26	*n* = 0
Total	*n* = 205	*n* = 86

### Image acquisition

Patients received a single injection of ^68^ Ga-PSMA (mean dose ± standard deviation, 130 ± 11 MBq, range 114–158 MBq) 60 min prior to image acquisition. To reduce tracer activity in the bladder, ureters, and kidneys, furosemide was injected intravenously 30 min prior to the radiotracer injection (0.13 mg/kg), patients drank 200 mL of water prior to radiotracer injection, and patients were asked to void prior to the scan. PET/CT acquisitions were performed on a Discovery VCT 690 PET/CT (GE Healthcare) (*n* = 38) or on a Discovery MI PET/CT (GE Healthcare) (*n* = 29) with six-bed positions (2.5 min acquisition time). CT as part of PET/CT was performed using the following scan parameters: tube voltage 140 kVp, tube current with automated dose modulation of 80 mA/slice, collimation 512 × 0.976, pitch 1.0, rotation time 0.5 s, coverage speed 78 mm/s, field of view (FOV) 50 cm. Images with a transverse pixel size of 1.00 and a slice thickness of 1.25 mm were reconstructed in the axial plane using the standard kernel.

### Image segmentation and radiomic feature extraction

**Fig. 2:**
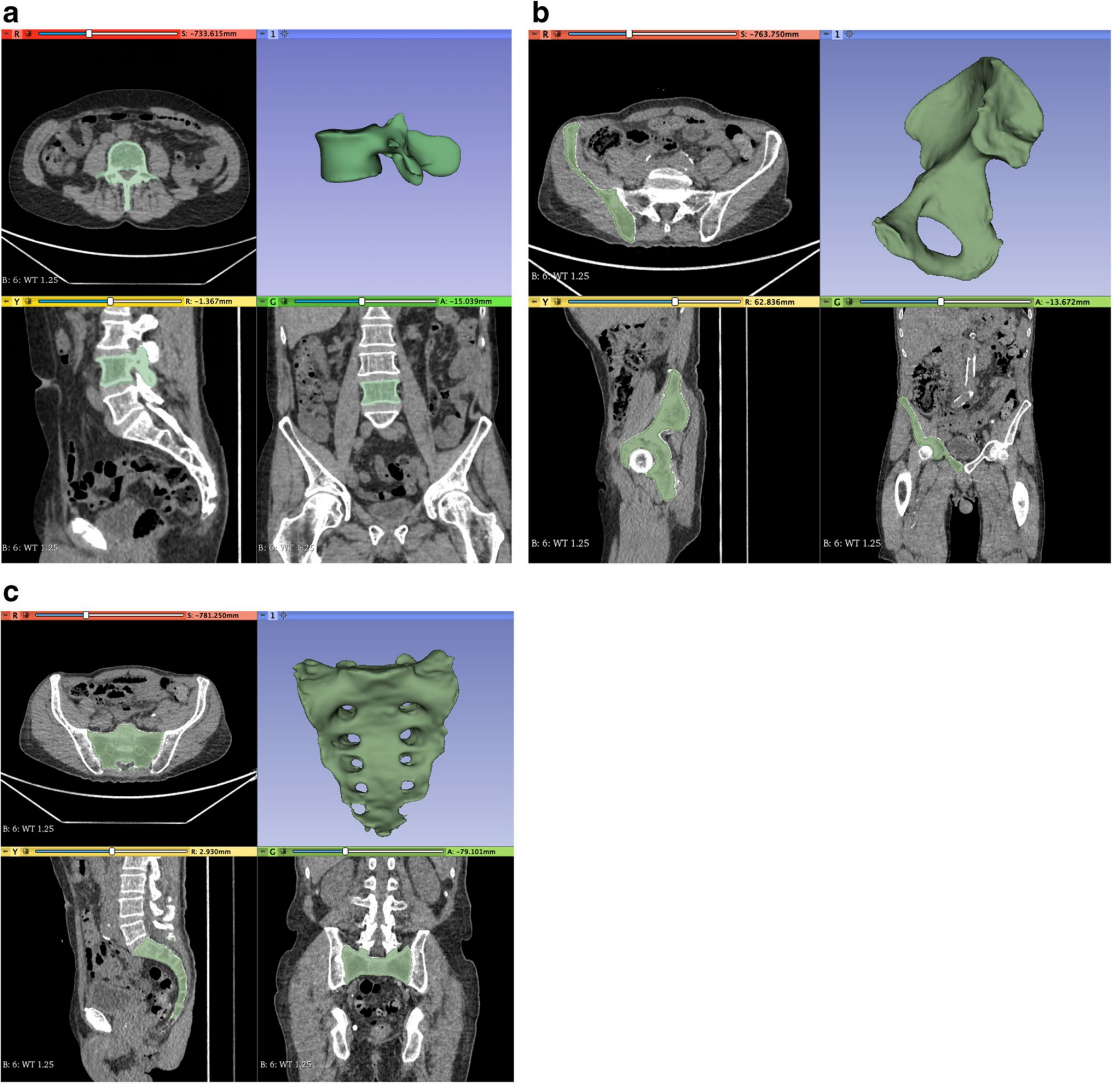
Representative examples of 3D bone volume segmentations of L4 (**a**), the right iliac bone (**b**), and the sacrum (**c**)

Image segmentation was performed semi-automatically with a commonly used open-source software platform (3D Slicer, Version 4.11; www.slicer.org; [19]) by two independent and blinded readers (with one and 4 years of experience in oncologic radiology, respectively) (Fig. 2). After loading the digital imaging and communications in medicine (DICOM) files (complete set of CT images), the entire bone (e.g., whole vertebra or entire iliac bone) with a ^68^ Ga-PSMA-positive bone metastasis (total of 205 metastases in the 67 patients) was manually segmented using the standard segment editor tool (Fig. 2). In addition, bone regions of the thoracic/lumbar spine and the pelvic bones appearing normal on PET/CT were manually segmented in each patient (total of 86 normal bones in the 67 patients) in order to investigate whether a radiomics classifier is a feasible option in the categorization of metastases vs. no metastases of all segmented bone volumes.

Before feature extraction, segmented images were pre-processed in order to minimize the influence of contrast and brightness variations on texture features [20, 21]: Images were spatially resampled to 2 × 2 × 2 mm using sitkBSpline as SimpleITK constant; signal intensity values were discretized to a bin width of 25 with relative intensity rescaling. The voxelArrayShift was set to 1000. All segmented bone volumes were separately included in the analysis. Subsequently, a total of 1218 radiomic features were extracted using the open-source tool pyRadiomics [21] integrated as a plugin into 3D Slicer. Extracted radiomic features comprised 5 different categories: histogram, gray-level cooccurrence matrix (GLCM), gray-level run length matrix (GLRLM), gray-level size zone matrix (GLSZM), and gray-level dependence matrix (GLDM). On each feature matrix, additional wavelet filtering (8 decompositions per level) and 5 different Laplacian of Gaussian filters (with sigma values of 1, 2, 3, 4, and 5) were applied. Shape features were not extracted, since only entire bones were segmented.

### Analysis of intra- and inter-reader reproducibility

To determine the intra-reader reproducibility, the first reader (with 1 year of experience in oncologic radiology) repeated the segmentation of 10 randomly selected subsets after a pause of 4 weeks and in random order. The second reader (with 4 years of experience in oncologic radiology) segmented the same image volumes to determine the inter-reader reproducibility.

### Data augmentation

To account for slight imbalances in the dataset (imbalanced ratio: 0.42), we performed a data augmentation step in order to achieve improved class balance and to avoid model overfitting. Data augmentation was performed using the imbalance package [22] in R (R Foundation; version 3.4.0) [23] and applying a Majority Weighted Minority Oversampling Technique (MWMOTE). After applying the MWMOTE technique, the dataset consisted of an equal number of observations with bone metastasis (*n* = 205) and normal bone (*n* = 205).

### Splitting of the dataset into training and testing datasets

In order to ensure the generalizability of the trained statistical models, the balanced dataset was then randomly split into separate training (*n* = 328 observations, *n* = 164 bone metastases and *n* = 164 normal bones) and testing datasets (*n* = 82 observations, *n* = 41 bone metastases, and *n* = 41 normal bones) using a ratio of 0.8:0.2. The entire dimension reduction and feature selection process described as part of the results section was performed only on the training dataset.

### Statistical analysis, dimension reduction, and feature selection

Statistical analyses were performed in R (R Foundation; version 3.4.0) using R Studio (RStudio; version 1.0.136 [24]). All continuous data are given as mean ± standard deviation (SD). Categorical variables are expressed in percent.

Intra- and interobserver agreement of radiomic features was assessed by calculating intraclass correlation coefficients (ICCs). A cut-off of 0.90 was used for selecting highly reproducible features.

**Fig. 3:**
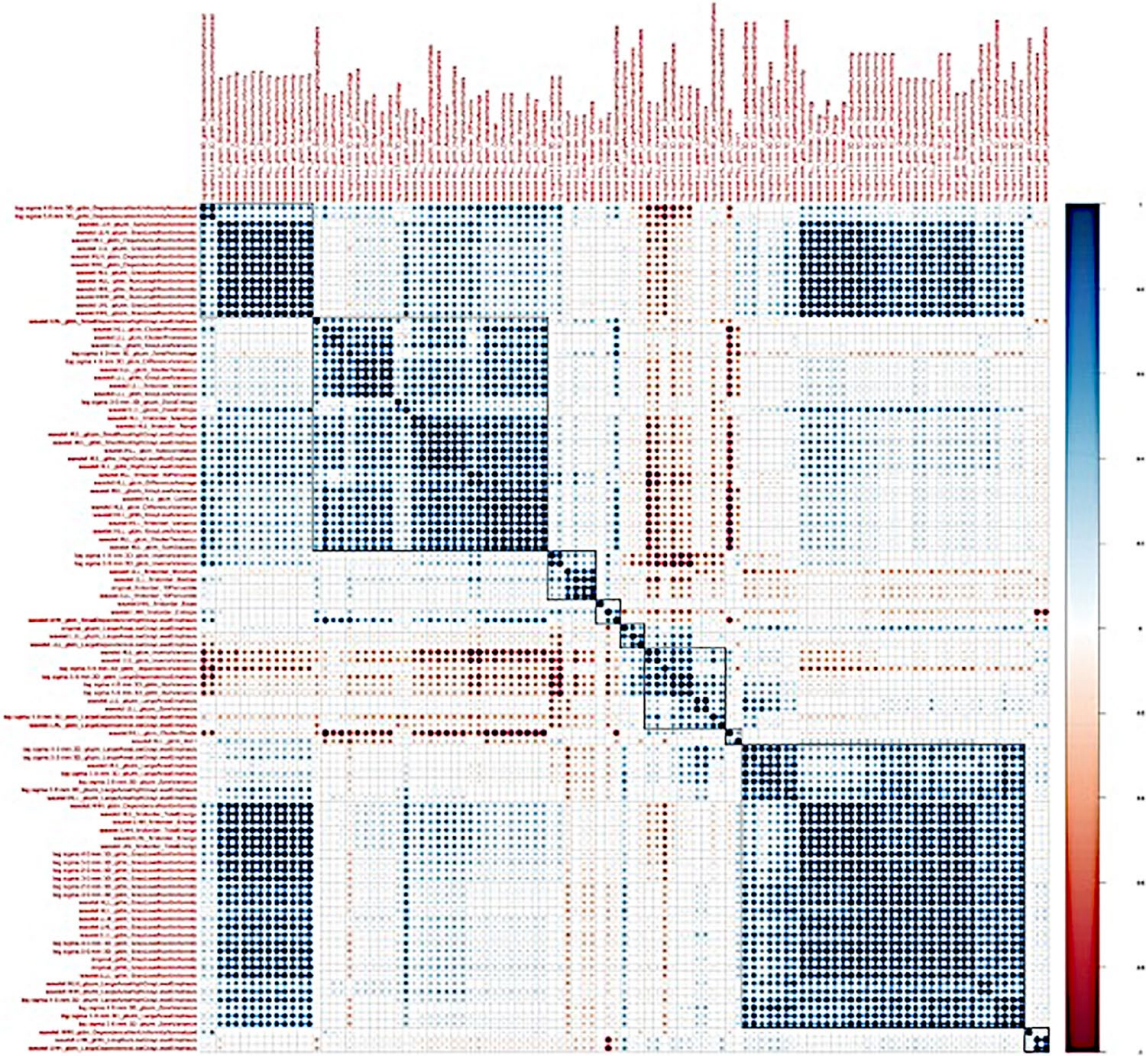
Correlogram illustrating the auto- and cross-correlation of the 105 most important features to classify metastatic and normal bone. Features were recorded after hierarchical clustering for depicting different feature clusters. Eleven clusters of radiomic features were identified (rectangular boxes). Blue points indicate positive correlation, red points negative correlation. The larger the points and the darker the color, the higher the correlation between two variables

Radiomics feature selection and dimension reduction were performed on the augmented training dataset only. In a first step, a total of 367 out of 1218 features were excluded based on calculation of intra- and interobserver reproducibility, with ICCs < 0.90 considered as non-reproducible. After normalization of all features using Z-score standardization, the remaining 851 features were fed into the Boruta dimension reduction and feature elimination algorithm as previously described [25, 26], resulting in selection of 105 features, which were considered most important for classification accuracy (Supplemental Fig. 1). Since the Boruta algorithm does not account for collinearity in the data, a correlation matrix was calculated in a next step in order to detect clusters of highly correlated features (defined as Pearson’s *r* ≥ 0.60; Fig. 3). After fitting separate random forest models on each of the 11 detected correlation clusters, only one feature from each cluster with the highest mean decrease accuracy index was selected for further analyses.

## Results

At the end of the multi-step dimension reduction process, the following 11 most important and independent features were selected for further statistical analyses: log.sigma.5.0.mm GLDM dependence non-uniformity normalized, wavelet-LHL-transformed GLSZM gray-level non-uniformity (GLN), log.sigma.4.0.mm GLSZM zone percentage, log.sigma.5.0.mm GLCM inverse variance, wavelet-HHL-transformed first-order mean, wavelet-LHH-transformed first-order entropy, wavelet-LLL-transformed GLSZM large-area low gray-level emphasis (LALGLE), wavelet-HHH-transformed GLSZM large-area high gray-level emphasis (LAHGLE), wavelet-HLL-transformed GLCM cluster shade, log.sigma.5.0.mm GLDM dependence variance, and wavelet-LHH transformed GLDM large dependence low gray-level emphasis (LDLGLE).

### Training of a radiomics-based machine learning classifier

**Fig. 4:**
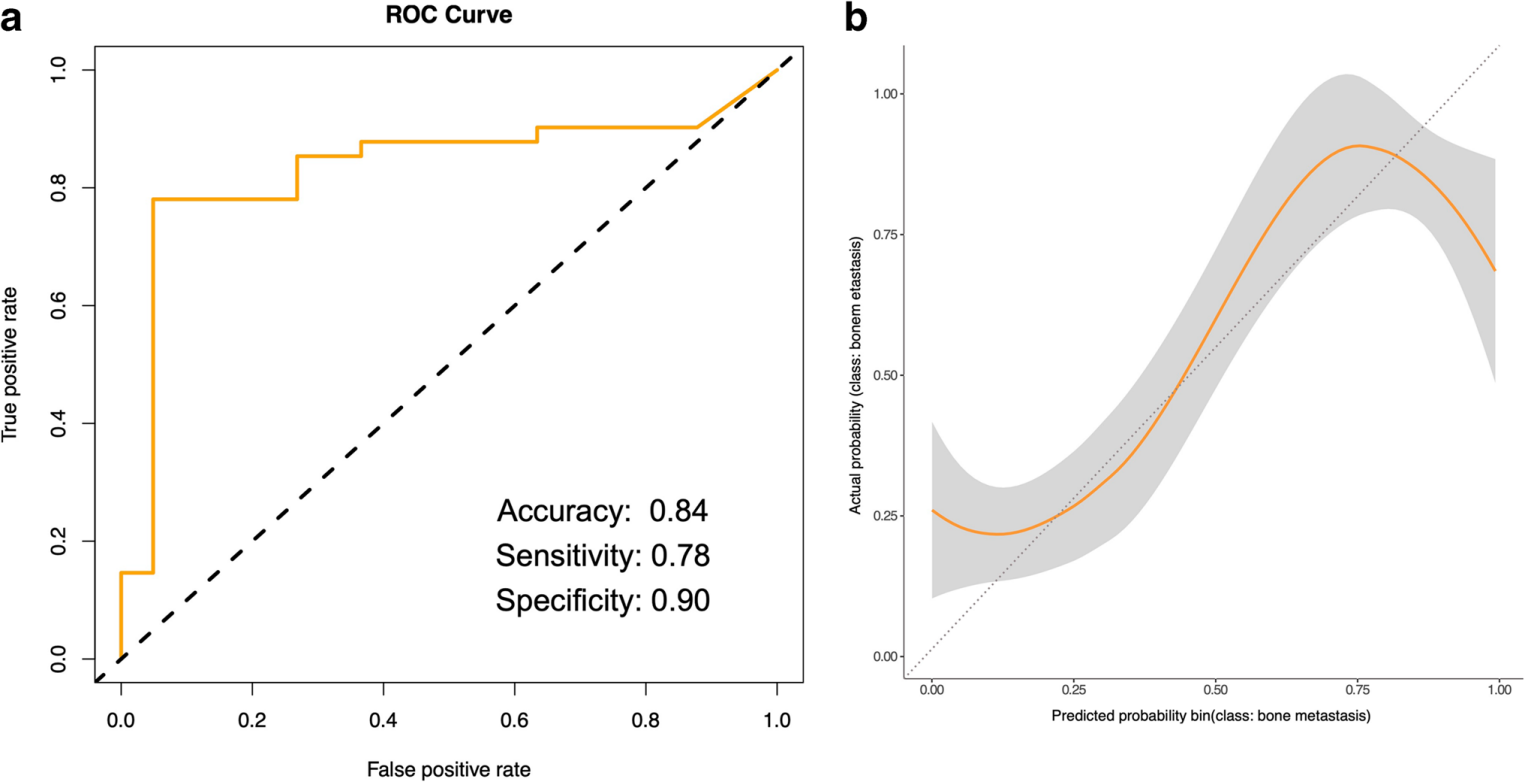
Graph represents receiver operating characteristic (ROC) analysis (**a**) and the calibration plot (**b**) for the trained machine learning algorithm in order to differentiate between bone metastases and normal bone. The ROC analyses indicate accuracy, sensitivity, and specificity of the gradient-boosted tree trained on the selected 11 most important radiomic features and applied on the independent test dataset. The calibration plot shows the calibration in terms of agreement between the predicted and the actual probability of bone metastases

A gradient-boosted tree was trained on the selected 11 radiomic features in order to classify patients’ bones into bone metastasis and normal bone using the training dataset. The model was tuned, leading to the following optimal tuning parameters: nrounds = 50, max_depth = 3, eta = 0.4, gamma = 0, colsample_bytree = 0.8, min_child_weight = 1, subsample = 0.75. Leave-one-out cross-validation was used to validate the model’s performance on the independent test dataset, which had not been shown to the algorithm before. Since the dataset was relatively small, this approach ensures that the greatest amount of data was used for each round of training of the model. In addition, this approach avoids the randomness of splits, since the model is trained on every possible combination of observations. The trained model achieved a classification accuracy of 0.84 (95% confidence interval [CI]: 0.74–0.91, *p* < 0.001) with 78% sensitivity and 90% specificity in the test dataset (Fig. 4a). The calibration plot (Fig. 4b) indicates some model overfitting. Further feature reduction did not lead to any additional improvement of the model.

## Discussion

In our study, we assessed the potential of a CT-radiomics-based machine learning approach to enable the discrimination between metastatic and metastasis-free bone matrix in patients with PCa, while using ^68^ Ga-PSMA PET/CT as the reference standard. The main finding of our proof-of-concept study indicates that the usage of a gradient-boosted tree, trained on the selected 11 most important CT-derived radiomic features, achieved a diagnostic accuracy of 90% with 91% sensitivity and 88% specificity.

We decided to use a gradient-boosted tree to train our model. Gradient boosting is a technique from the ensemble learning spectrum, which—instead of finding the optimal hyperparameters for a single model—uses several complementary weak models to build a more powerful ensemble model. For multidimensional datasets such as in radiomics, ensemble learning techniques have been shown to be powerful techniques with higher accuracies [16]. The drawback is the higher tendency to overfitting, especially in smaller sample sizes.

Particularly small lesions within the bone marrow without substantial destruction of the bone matrix can be missed with CT [27]. Likewise, technetium 99 m (^99m^Tc) diphosphonate bone scan shows only a limited detection rate of bone metastases, particularly of small lesions with low bone turnover [27]. A meta-analysis of 1102 patients with PCa comprising 12 studies using ^99m^Tc bone scan showed a sensitivity of only 59% and a specificity of 75% for bone metastasis detection [28]. Since both bone scan and CT will underestimate the presence and extent of metastatic bone disease, PET imaging is increasingly used in these patients, often resulting in upstaging of the disease [27, 29, 30].

**Fig. 5:**
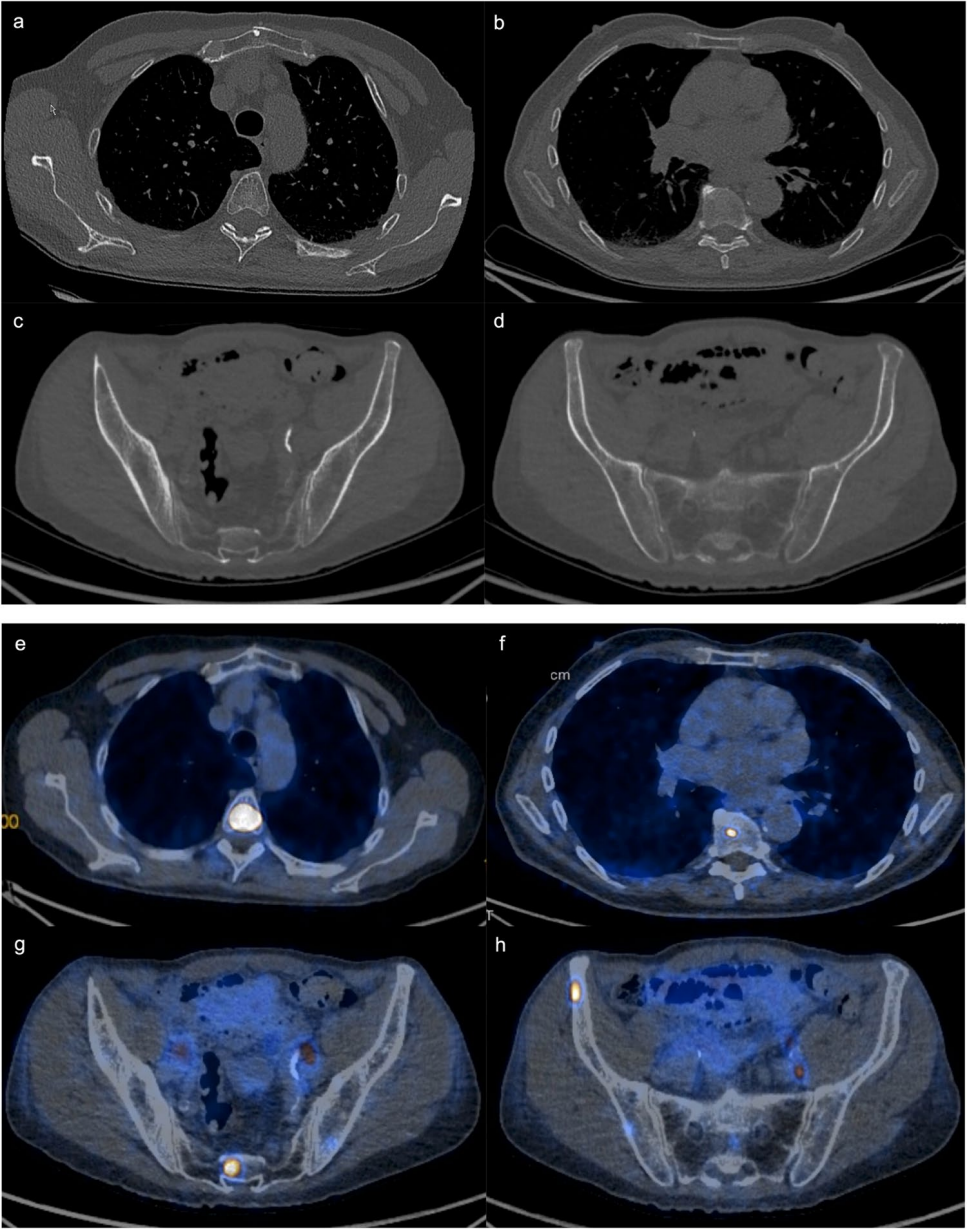
CT and corresponding PET/CT in three representative patients with metastatic bone disease from PCa. Can you identify the bone metastases in the upper (**a**) and mid (**b**) thoracic spine, the inferior part of the sacrum (**c**), and the right iliac bone (**d**) on CT only, without the additional metabolic information from PET? Corresponding PET/CT images clearly show high ^68^ Ga-PSMA uptake of the bone metastases in the aforementioned skeletal regions (**e**–**h**). Note additional ^68^ Ga-PSMA-positive lymph node metastases along the left iliac vessel axis (**g**, **h**)

According to the results of our proof-of-concept study, CT imaging as an inexpensive, easily accessible, and time-saving modality might be a feasible option to differentiate between bone metastases and metastasis-free bone in patients with metastatic PCa, showing an overall good diagnostic accuracy in this moderately large patient group, hereby displaying image information that may not be visible for the radiologists’ eye (Fig. 5). It has to be noted, however, that the trained model showed some overfitting when considering the calibration plot. This was somewhat expected due to the relatively low lesion number included in this proof-of-concept study. As a consequence, future studies should retrain the model on a considerably larger dataset.

Recently, a study by Acar et al. [10] evaluated the potential differences between sclerotic bone lesions with ^68^ Ga-PSMA uptake (metastatic) and those without (completely responded) using CT texture analysis and machine learning. Similar to our study (bone metastasis vs. metastasis-free bone), the visual distinction between metastatic and completely responded sclerotic bone lesions on CT was not possible in their study. However, they detected statistically significant differences between both groups in 28 of 35 acquired texture features, resulting in an overall good accuracy of 73.5% [10].

Another study by Xu et al. developed a texture analysis and machine learning method on CT- and ^18^F-fluorodeoxyglucose (FDG) PET images to differentiate between malignant and benign bone and soft tissue lesions [31]. While the accuracy of CT-derived texture features was separately calculated with 73% (sensitivity 81% and specificity 61%), the accuracy increased to 83% (sensitivity 86% and specificity 77%) when combined with the additionally acquired PET texture features. Xu et al. [31] included several different entities of benign and malignant bone lesions in the analysis, representing a possible explanation for the slightly lower performance compared to the results of our study.

The main purpose of our study was to address the diagnostic and clinical dilemma of frequently missing metastatic bone disease in CT, which can only be detected with additional information of PET imaging due to the lack of morphologic changes. From this clinical point of view, we had to perform the segmentation of the entire bone of a metastatic lesion (e.g., whole vertebra), in order to use the radiomic classifier for the categorization in metastases vs. no metastases. In contrast to our study, Acar et al. [10] and Xu et al. [31] performed a preselection by including only the target lesions in the volume of interest, suggesting even greater value of our results. Despite the merely good diagnostic accuracy of our study with an accuracy of 0.85 (sensitivity 78% and specificity of 90%), it can be assumed that the human eye (without the metabolic information) would hardly be able to detect these relatively small lesions without or only subtle morphologic changes, although our study is lacking the comparison with a human reader.

Besides the early detection of metastatic bone disease, a timely and accurate prediction of bone metastases and identification of patients at high risk for bone metastases would be highly desirable and could allow for the selection of those patients most likely to benefit from targeted therapy. Recently, Wang et al. [32] developed and validated an MRI-based radiomics model for the individualized pretreatment prediction of bone metastases in patients with PCa. T2w and dynamic contrast-enhanced (DCE) T1w images in combination with clinical risk factors showed excellent predictive performance in the training cohort with an area under the curve (AUC) of 0.92 (accuracy: 0.85; sensitivity: 0.81, specificity: 0.89), which could be confirmed in the validation cohort (AUC = 0.9; accuracy: 0.85; sensitivity: 0.82, specificity: 0.88). Zhang et al. [33] confirmed these results in their study, additionally incorporating the DWI sequence in the newly developed MRI-based radiomics nomogram reaching an AUC of 0.93 in the training cohort and an AUC of 0.92 in the validation cohort, introducing a robust model for predicting bone metastases in patients with newly diagnosed PCa. We believe that the results of our study could add incremental value for diagnostic and treatment strategies, especially in patients with high probability of bone metastases according to the aforementioned MRI-based radiomics nomogram.

The currently favored PSMA radiotracer is ^18^F-PSMA, and not anymore ^68^ Ga-PSMA, which was used in our study. Besides having several advantages over ^68^ Ga-PSMA, such as less noise, mainly owing to lower positron energy and higher positron yield, and less activity in the urinary bladder, ^18^F-PSMA has the major disadvantage of occasional unspecific bone uptake [34, 35]. This uptake is supposedly due to defluorination and is typically seen in the ribs and pelvic bone, which are predilection sites for prostate cancer bone metastases [34, 35]. Hence, CT-based radiomics might be helpful in clinical ^18^F-PSMA scans in order to differentiate unspecific PSMA uptake from bone metastases.

Although radiomics has demonstrated its potential for diagnostic, prognostic, and predictive purposes, the method is still facing certain challenges. The reproducibility of radiomics studies is often poor, which is partly due to the retrospective nature of most studies resulting in insufficient standardization of imaging protocols, including acquisition and reconstruction parameters [36]. Accordingly, it has been shown that automatic image segmentation enhances reproducibility of texture features and thus would be the preferred technique to improve standardization of radiomics analyses [36]. Also, the lack of adequate validation with the consequence of statistical type I errors impedes the transition to routine clinical practice [37]. Furthermore, the reproducibility of radiomic features might also depend on different modalities or scanners and is not necessarily generalizable to various disease entities [38].

Adding to the aforementioned shortcomings of the applied methodology, we acknowledge several other limitations of our study. First, there were the inherent drawbacks of the retrospective study design. Second, pelvic bone and vertebral bodies without ^68^ Ga-PSMA uptake were considered unaffected, metastasis-free bone. Third, our dataset consisted of paired data with different observations from the same patient. Since it was not possible to correct for this potential bias during the training of our model (due to methodological constraints), this might have led to potential overfitting of our model. Fourth, no clinical parameters were included in the radiomics analysis, although this might have the potential to further increase the diagnostic accuracy. Fifth, no external validation dataset was used. Another limitation is the use of different PET/CT scanners with different intrinsic system sensitivity.

In conclusion, our proof-of-concept study shows promising results of radiomics applied on CT images for the differentiation between bone metastases and metastatic-free bone in patients with PCa. Importantly, radiomics enabled this differentiation in a quantitative way on CT images showing only discrete abnormality. Future advancing applications include fully automatically bone segmentation frameworks for all patients with newly diagnosed prostate cancer, followed by the usage of a radiomics classifier, allowing for an opportunistic screening-like approach in the early detection of bone metastases.

## Supplementary Information

Below is the link to the electronic supplementary material.

Supplementary file1 (DOCX 227 KB)

## Abbreviations

AUC: Area under the curve
CT: Computed tomography
DCE: Dynamic contrast enhancement
DICOM: Digital Imaging and Communications in Medicine
FDG: Fluorodeoxyglucose
Ga: Gallium
GLCM: Gray-level coccurrence matrix
GLDM: Gray-level dependence matrix
GLRLM: Gray-level run length matrix
GLSZM: Gray-level size zone matrix
LAHGLE: Large-area high gray-level emphasis
LALGLE: Large-area low gray-level emphasis
MBq: Mega Becquerel
MRI: Magnetic resonance imaging
MWMOTE: Majority weighted minority oversampling technique
PCa: Prostate cancer
PET: Positron emission tomography
PSA: Prostate-specific antigen
PSMA: Prostate-specific membrane antigen
ROC: Receiver operating characteristic
SD: Standard deviation
Tc: Technetium

## Declarations

### Guarantor

The scientific guarantor of this publication is Prof Hatem Alkadhi.

### Statistics and biometry

One of the authors has significant statistical expertise.

### Methodology

• retrospective

• performed at one institution
