# Supplementary material for: Radiomics for detecting prostate cancer bone metastases invisible in CT: a proof-of-concept study
Source: Eur Radiol. 2021 Sep 24;32(3):1823–32. doi: 10.1007/s00330-021-08245-6 (PMC8831270; doi:10.1007/s00330-021-08245-6)

**Supplemental Figures**

**Supplemental Figure 1:** Graphical output of the Boruta radiomic feature selection and dimension reduction process for differentiating bone metastases from unaffected bone, showing importance measures (mean decrease accuracy) for each input variable above the shadowMin. Green and red boxplots indicate Z-scores of confirmed and rejected variables, respectively. Blue boxplots indicate the minimal, average, and maximum Z-score of a shadow attribute.


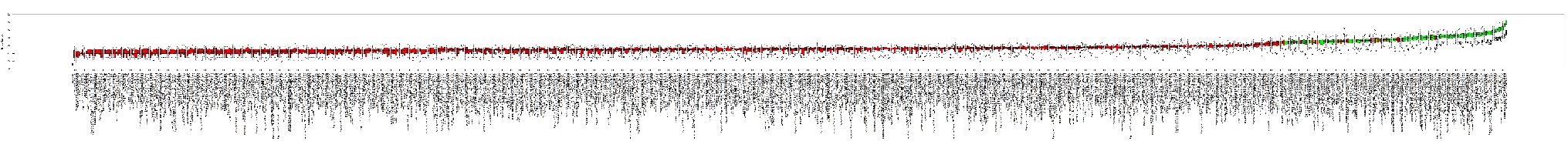

Supplement: Supplementary file 1 — Supplementary file1 (DOCX 227 KB) [file 330_2021_8245_MOESM1_ESM.docx]
